# An Elderly Gentleman with Acute Lupus Pneumonitis as the Initial Manifestation of Systemic Lupus Erythematosus

**DOI:** 10.1155/2021/2692735

**Published:** 2021-07-24

**Authors:** Gina Ferrero, Kate Chernow, Marissa Karpoff, Pamela Traisak, David Feinstein, Hala Eid

**Affiliations:** ^1^Cooper University Hospital, Camden, NJ, USA; ^2^Einstein Medical Center Montgomery, East Norriton, PA, USA; ^3^Abington Lansdale Hospital, Lansdale, PA, USA

## Abstract

Systemic lupus erythematosus is a systemic autoimmune disease, with presentations that vary within a population and across the lifespan of an individual. The disease afflicts childbearing women more than men and uncommonly presents in the geriatric population. Lupus pneumonitis is rare, with a reported incidence of 1–4%. Herein, we discuss the case report of an elderly gentleman with biopsy-proven acute lupus pneumonitis (ALP) as an initial presentation of lupus. After starting high-dose steroids, the patient initially improved, though unfortunately endured a non-ST elevation myocardial infarction and recurrent gastrointestinal bleeding. Despite multiple interventions and a prolonged hospital course, his gastrointestinal bleeding persisted. He elected to go on home hospice and ultimately passed away due to ongoing gastrointestinal bleeding. As with our patient, elderly patients can pose a diagnostic dilemma with regard to late-onset lupus; multiple comorbidities and growing evidence that late-onset lupus may manifest with distinct clinical patterns from younger cohorts complicate diagnosis in these patients. It is critical to maintain a broad differential, which includes unusual rheumatic manifestations when management of common comorbidities fails to alleviate symptoms for an elderly patient. Failure to do so may result in delayed diagnosis of rheumatic disease and increased side effects related to treatment. Additionally, this case serves as a reminder that due to the complexity of rheumatic disease and the additional challenge of older patients with baseline comorbidities, sometimes palliative care options may be appropriate.

## 1. Introduction

Systemic lupus erythematosus (SLE) is a chronic, systemic autoimmune disease that is characterized by disease “flares” and immunologic abnormalities such as antinuclear antibodies. The disease more frequently plagues women of childbearing age than men or elderly. SLE is an enigma, in that we have not fully elucidated all genetic and environmental triggers that lend to the disease. Nevertheless, the typical clinical manifestations, which may vary dramatically from patient to patient, are well-described. We discuss an unusual case of SLE manifesting with acute lupus pneumonitis in an elderly gentleman. Elderly onset lupus is rare; moreover, an initial presentation of lupus pneumonitis is less common but does occur [1]. Of the lupus pulmonary manifestations, lupus pneumonitis is rare even across all demographics with an incidence of 1–4% [2, 3].

## 2. Case Description

The patient is an 86-year-old male farmer who presented with several months of intermittent nonmassive hemoptysis and a one-week history of progressively worsening dyspnea and fatigue. The patient's medical history was significant for recently diagnosed transfusion-dependent myelodysplastic syndrome, coronary artery disease requiring stenting, atrial fibrillation, and interstitial lung disease attributed to amiodarone requiring 2 L/min oxygen at home. His home medications were pantoprazole 40 mg daily, zolpidem 5 mg nightly, and furosemide 20 mg daily. His hemoptysis had persisted despite discontinuing his dual antiplatelet therapy for his coronary stents. Additionally, the patient's anemia required frequent transfusions to an extent which did not correlate with the mild degree of myelodysplastic syndrome. Therefore, further investigation was warranted.

**Figure 1:**
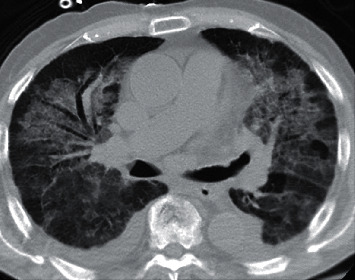
CT chest demonstrating diffuse ground glass opacities bilaterally, central predominant, no focal infiltrates.

On the initial physical exam, our patient was afebrile and hemodynamically stable with an oxygen saturation of 94% on 2 L/min oxygen. Breath sounds were notable for fine bibasilar crackles. He exhibited bilateral lower extremity pitting edema. Exam was otherwise unremarkable. Basic laboratory tests were significant for leukocytosis with a left shift noted with a white blood count 21.14 10^*∗*^3/uL (4.5–11^*∗*^3/uL) and a microcytic anemia noted with a hemoglobin level 7.8 g/dL (14–18 g/dL). He had a mildly elevated creatinine to 1.31 mg/dl (0.6–1.2 mg/dl). NT-proBNP was 5,375 pg/ml (0–852 pg/ml). Erythrocyte sedimentation rate was greater than 100 mm/hr (0–15 mm/hr), and C-reactive protein was 23.21 mg/dl (<0.5 mg/dl). Imaging with computed tomography (CT) chest demonstrated bilateral diffuse ground glass opacities with central predominance and a mild right-sided effusion (Figure 1).

He was initially treated with diuresis, as there was concern that his pulmonary findings were related to volume overload in the setting of a recent blood transfusion. He was also treated empirically with broad spectrum antibiotics. Despite these efforts, our patient's respiratory status progressively worsened with increasing oxygen requirements to 6 L/min nasal cannula. Arterial blood gas was collected at that time which showed significant hypoxia with arterial PO_2_ 38 mm (Hg) (86–100 mm (Hg)). Due to his clinical presentation and progressive symptoms, further workup was warranted. A rheumatologic workup was completed. When questioned, the patient endorsed late-onset Raynaud's symptoms, occasional arthralgias without swelling or morning stiffness, and oral sicca symptoms. He denied any history of alopecia, photosensitivity, rashes, oral or nasal ulcers, skin thickening, dysphagia, or reflux disease. He noted that his sister has SLE. Laboratory tests were significant for positive antinuclear antibodies with a 1 : 160 titre in homogenous pattern, C4 complement was decreased to 14 mg/dL (20–59 mg/dl), and C3 complement was normal. Anti-DNA was elevated at 194 IU/ml (<100 IU/ml). Anti-Ro (SSA) was positive at 6.0 (<1.0) and anti-La (SSB) was positive at >8.0 (<1.0). Other rheumatological laboratory tests were negative including anti-Smith antibody, anti-RNP antibody, antichromatin, proteinase 3 antibody, myeloperoxidase antibody, and glomerular basement membrane antibody.

**Figure 2:**
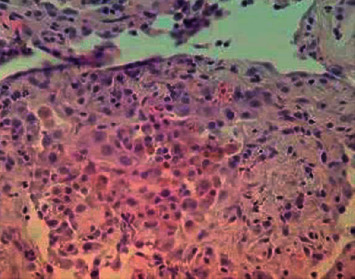
Lung parenchyma showing chronic hemorrhage (hemosiderin macrophages), septal acute inflammatory cells with thickening of the alveolar septae, and type II pneumocyte hyperplasia.

Our patient underwent bronchoscopy and transbronchial biopsy. The bronchoscopy and bronchoalveolar lavage (BAL) showed no findings of diffuse alveolar hemorrhage (DAH) on lavage aliquots. BAL demonstrated 165 cells/uL, 85% segmented neutrophils, 1% lymphocytes, 8% mono/macrophages, and 4% eosinophils. Blood cultures and BAL cultures, including fungal cultures, were negative for infection. The BAL acid-fast bacillus culture grew no acid-fast organisms after six weeks. Cytology was negative for malignant cells. Transbronchial biopsies showed chronic hemorrhage (hemosiderin-laden macrophages), septal acute inflammatory cells with thickening of the alveolar septae, and type II pneumocyte hyperplasia consistent with capillaritis (Figure 2). Given lack of exposure to medications known to cause capillaritis (such as hydralazine, propylthiouracil, phenytoin, retinoic acid, or illicit drugs) in combination with positive ANA, low C4, and high titre anti-DNA, our patient was diagnosed with acute lupus pneumonitis.

## 3. Treatment and Outcome

Our patient was started on methylprednisolone 60 mg IV daily for acute lupus pneumonitis. Within twenty-four hours of initiating steroids, the patient's respiratory status improved; he was able to converse without dyspnea and was able to ambulate short distances with 2 L/min nasal cannula. Unfortunately, a few days later, he suffered a non-ST elevation myocardial infarction (NSTEMI) for which he underwent cardiac catheterization and coronary stenting. He started back on antiplatelet therapy. The hospital course was further complicated by an upper gastrointestinal bleed secondary to a duodenal ulcer resulting in hypotension requiring vasopressors. Methylprednisolone had to be discontinued temporarily until his gastrointestinal bleed was controlled. Methylprednisolone was ultimately restarted after which the patient endured additional gastrointestinal bleeding. Nevertheless, antiplatelet therapy was continued given the high risk for in-stent thrombosis. Over the course of three weeks, our patient required three esophagogastroduodenoscopies with clipping as well as one coil embolization at the gastroduodenal artery and a later coil embolization at the pancreaticoduodenal artery. In light of stressful repeat procedures and a prolonged hospital course, the patient and family elected to focus on comfort. In the case of further gastrointestinal bleeding, he did not desire further invasive procedures. His goal was to be with his family. He ultimately passed away six days after hospital discharge due to recurrent gastrointestinal bleeding in the company of his loving family.

## 4. Discussion

Lupus pneumonitis, one of several respiratory complications of SLE, is rare, with a reported incidence of 1–4% [2, 3]. Furthermore, lupus pneumonitis is uncommon as an initial presentation of SLE; a registry report released in 2018 of 3,215 lupus patients showed only 4% of cases had pulmonary disease of any kind upon initial presentation of SLE, with rates of lupus pneumonitis likely much lower [2]. It should be noted, however, that for late-onset SLE (lupus diagnosed after the age of fifty), the incidence of pulmonary manifestations appears to be increased compared to the younger population (pulmonary involvement (21.2% vs. 11.3%) [4]. Therefore, despite its rarity, lupus pneumonitis as an initial presentation of SLE in the elderly does occur (in this case and in others [1]) and warrants diagnostic consideration.

The constellation of symptoms suggestive of acute lupus pneumonitis is as follows: dyspnea, pleuritic chest pain, cough, fevers, and less occasionally, hemoptysis. [5–7] Hypoxemia is invariably present. Bloodwork is typically significant for high anti-DNA titres [5, 7]. As exemplified in our patient, many previously described patients with lupus pneumonitis have anti-SSA antibodies (up to 82%) [7–9]. Radiographically, we see diffuse ground glass opacities and areas of consolidation suggestive of acinar filling. These findings tend to be in the bilateral lower lobes with or without pleural effusion [5, 10]. As a diagnosis of exclusion, lupus pneumonitis requires evaluation by bronchoscopy with BAL. BAL is primarily used to exclude infectious etiologies and DAH. Biopsy is rarely required to confirm diagnosis.

Historically, there has been debate about the histopathology of lupus pneumonitis and even its existence. Complicating this, the pathological finding of pulmonary fibrosis to suggest acute or chronic lupus pneumonitis is infrequent, though clinical descriptions of lupus pneumonitis are more easily found in literature [11]. One necropsy series explained each case of clinically diagnosed lupus pneumonitis by other processes such as infection, aspiration, heart failure, or uremia [12]. It is important to emphasize that our patient fit not only the clinical picture of lupus pneumonitis but also had consistent biopsy characteristics without another explanation. Both diffuse alveolar damage (DAD) and capillaritis were evident in our patient. DAD refers to hyaline membranes with cellular interstitial infiltrates that result in alveolar edema. Capillaritis occurs in the setting of complement and immunoglobulin protein deposition and characteristic neutrophilic infiltrates in the alveolar interstitium. The process results in injury and necrosis of capillary walls, disruption of the epithelial-endothelial alveolar basement membrane, and subsequent extravasation of neutrophils and red blood cells into the alveolar space. As demonstrated in our patient, hemosiderin-laden macrophages secondary to erythrophagocytosis and interstitial hemosiderin implied a chronic pathological process [13].

The diagnosis of lupus pneumonitis was not initially at the top of our differential, particularly given the patient's demographic and comorbidities. He was initially treated preemptively for volume overload and an infectious process. Unique aspects of the case bring to mind additional differentials. The patient's occupation as a farmer makes subacute hypersensitivity pneumonitis a potential cause, though such a patient would not present with hemoptysis. CT chest in this case would be expected to show centrilobular nodules, mid-to-upper lobe predominant ground glass opacities, and evidence of air trapping. In a patient with myelodysplastic syndrome, paraneoplastic autoimmune syndromes may be considered. Regarding the patient's history of amiodarone-induced interstitial lung disease (ILD), we were unable to obtain records from the period of the diagnosis and therefore cannot comment on whether or not the patient presented in the typical manner. If the diagnosis of amiodarone-induced lung toxicity was not straightforward, the new diagnosis of lupus pneumonitis brings to light the possibility that the gentleman had chronic SLE-associated ILD, particularly given his autoantibodies specific for SLE and CT changes. Similarly, the presence of SSA and SSB antibodies raises the possibility of Sjögren's syndrome complicated by ILD. Ultimately, the patient's acute clinical decline in addition to biopsy findings were consistent with an acute process as opposed to an insidious ILD. Though biopsy is typically not required to diagnose ILD, one would expect to find chronic infiltrate such as lymphocytes or plasma cells, as opposed to acute inflammatory cells (as in our patient) and possibly fibrosis [7]. Ultimately DAD and capillaritis were found on biopsy, and given concomitant serology, a positive ANA, low C4, and high titre anti-DNA, our patient was diagnosed with acute lupus pneumonitis.

Once our biopsy was reviewed, DAH remained high on the differential. It is important to note that both DAD and capillaritis may be seen in DAH in addition to ALP. In fact, DAD and ALP may be thought of as two processes on the same disease spectrum [10]. Additionally, our patient's clinical presentation of hemoptysis in conjunction with ground glass opacities on CT scan makes DAH a consideration. Nevertheless, the case is most consistent with ALP given lack of hemorrhagic aliquots on BAL in addition to biopsy findings. While biopsy findings are not specific to ALP versus DAH, the pattern found in our patient supports ALP. ALP may be used in reference to DAD without hemorrhage, while DAH demonstrates gross alveolar hemorrhage and less commonly shows DAD [3, 10]. Though DAH may present with capillaritis, the common pattern is “bland” DAH (DAH without capillaritis), which was not our patient's finding [7, 10].

Given that acute lupus pneumonitis has been thought of as a mild form of the same respiratory disease process that includes DAH, it is not surprising that treatment is similar [10]. Unfortunately, there are no controlled studies for treatment. Rheumatologists are guided based on approach to other well studied complications of SLE such as nephritis, insights into treatment for acute interstitial pneumonia, and case reports. Once infection is ruled out in the setting of acute lupus pneumonitis with BAL, antibiotics may be discontinued and steroids, the mainstay of treatment, initiated. Prednisone is used at a dose of 1–1.5 mg/kg bodyweight/day [7, 14]. If there is significant tachypnea or hypoxemia warranting intensive care unit level of care, methylprednisolone pulse treatment with 1 g/day for 3 days should be administered. Steroid sparing immunosuppressive therapy should be used when feasible. Cyclophosphamide is commonly prescribed for patients with inadequate response to steroids [3, 7]. Alternatively, azathioprine, rituximab, and intravenous immune globulin have been used with variable response [7, 15–17]. The prognosis of lupus pneumonitis is poor. The mortality rate is estimated to be 40–50% during the acute episode. Of the patients who survive lupus pneumonitis, 50% will have persistent interstitial infiltrates and lung function abnormalities suggesting progression of chronic interstitial pneumonitis [17]. While it is promising that our elderly patient initially improved with methylprednisolone, he eventually succumbed to complications of various disease processes and their competing treatments.

It is important to note that the 2019 European League Against Rheumatism/American College of Rheumatology classification criteria for SLE does not include pulmonary manifestations outside of serosal involvement [18]. Thus, given our patient's lack of other clinical criteria of SLE, he does not fit classification criteria. While classification criteria are excellent at describing a homogenous group of individuals for research purposes, they often fall short for the diagnostic use in more atypical cases such as our patient. This case report echoes Boddaert et al.'s epidemiological findings regarding late-onset SLE [4]. Patients with late-onset lupus were significantly more likely to have pulmonary complications, whereas they were less likely to demonstrate a number of findings of the classification criteria including photosensitivity, alopecia, nephritis, and malar rashes [4]. A number of other studies have also found that malar rashes and photosensitivity are significantly less common in elderly onset disease, whereas sicca symptoms are more common [19–21].

This case is a reminder that severe organ and life-threatening initial presentations of lupus occur in the elderly population and that vigilance is necessary to recognize these cases, particularly in light of other comorbidities that may cloud atypical presentations. The heterogeneous nature of lupus presentations is further compounded by the variable signs noted in elderly cohort studies. Knowing this, it is important for clinicians to be on guard against allowing improbable presentations of SLE to slip out of their differentials. In this case, diagnosis of SLE was not the only challenge. High-dose steroids are difficult for elderly patients to tolerate. Methylprednisolone likely contributed to our patients NSTEMI and recurrent GI bleeding. Therefore, in cases of elderly lupus patients requiring steroids, a thorough risk and benefit discussion with the patient and family members is prudent.

## Data Availability

The data used to support the findings of this study are available from the corresponding author.
